# Music listening for anxiety relief in children in the preoperative period: a randomized clinical trial**^1^**

**DOI:** 10.1590/1518-8345.1121.2841

**Published:** 2016-12-19

**Authors:** Mariana André Honorato Franzoi, Cristina Bretas Goulart, Elizabete Oliveira Lara, Gisele Martins

**Affiliations:** 2 MSc, Assistant Professor, Departamento de Enfermagem, Universidade de Brasília, Brasília, DF, Brazil.; 3 Undergraduate student in Nursing, Universidade de Brasilia, Brasília, DF, Brazil.; 4 PhD, Adjunct Professor, Departamento de Enfermagem, Universidade de Brasília, Brasília, DF, Brazil.

**Keywords:** Pediatric Nursing, Surgical Procedures, Operative, Anxiety, Music, Randomized Controlled Trial

## Abstract

**Objective:**

to investigate the effects of music listening, for 15 minutes, on the preoperative anxiety levels in children undergoing elective surgery in comparison with conventional pediatric surgical care.

**Method:**

randomized controlled clinical trial pilot study with 52 children in the preoperative period, aged 3 to 12 years, undergoing elective surgery and randomly allocated in the experimental group (n = 26) and control group (n = 26). Anxiety was assessed in both groups by the application of the modified Yale Preoperative Anxiety Scale and measurement of the physiological variables, upon arrival and 15 minutes after the first measurement.

**Results:**

there was a statistically significant difference in preoperative anxiety between the two groups only in relation to the physiological variable, since the respiratory rate of preschool children in the experimental group reduced in the second measurement compared to the control group (p = 0.0453). The experimental group showed a statistically significant reduction in anxiety levels after 15 minutes of music listening (p = 0.0441), specifically with regard to the behavioral domains of activity, vocalization, emotional expression and apparent awakening state.

**Conclusion:**

music listening emerges as a potential nursing intervention for relief of preoperative anxiety in children undergoing surgical procedures. RBR-7mcr59.

## Introduction

Pediatric hospitalization is a significant event in the lives of children and their families and usually tends to be a traumatic and stressful event, as it implies deprivation in the affective, cognitive and leisure spheres, and it is even more intense when associated with surgical procedure or if it is the child's first hospital experience^1^. It is estimated that between 40% and 75% of children undergoing surgery experience anxiety and fear in the preoperative period^2^, which are manifested by psychological and physiological changes.

In addition to the need of physio-biological care, it is important that nurses are attentive of the psychological, emotional, social and cultural needs of children, issues often ignored by the health team, which focuses mainly on restoring the organ weakened by the surgical procedure than on providing humanized, atraumatic and holistic care^3^.

Musical intervention is a therapeutic resource that has been increasingly used in nursing care as a complementary therapy to promote relaxation, emotional and spiritual comfort, distraction, wellness sensation^4^ and relief of pain in hospitalized patients^5^. Despite the use of musical intervention by nursing, the therapeutic effectiveness of music has not yet been clearly established^6^. Specifically in relation to the use of musical intervention applied to children undergoing surgical procedures, there are few national studies^6^ ^-^ ^7^, with highlight to a clinical trial that evaluated the therapeutic effect of music on pain in the postoperative period in children undergoing cardiac surgery^7^.

Therefore, considering the issue of children undergoing surgical procedures, specifically the relevant prevalence of the clinical outcome of preoperative anxiety and the need for evidence for the use of musical intervention in the nursing care context, this study was designed aiming at investigating the effects of music listening, for 15 minutes, in the preoperative anxiety levels in children undergoing elective surgery in comparison with conventional pediatric surgical care.

## Method

This is an experimental, double-blind, parallel-group, randomized controlled clinical trial (RCT) pilot study, carried out from September 2014 to April 2015, in a Pediatric Surgical Unit at a public hospital, reference in the care of children affected by surgical diseases in the Federal District and Surrounding Areas. The Research Ethics Committee of the Foundation for Education and Research in Health Sciences approved the trial, under protocol number 525.251, registered in the Brazilian Clinical Trials Registry database, which followed all the CONSORT guidelines (*Consolidated Standards of Reporting Trials*), specifically the extension to non-pharmacological treatment studies^8^.

Pre-school children (3-6 years) and schoolchildren (6-12 years), of both genders, undergoing elective surgery were included. The rationale for selection of children at these ages was based on prior knowledge of the age profile of the surgical unit demand, which was very variable. Therefore, it was necessary to consider such age range in order to enable data collection, in addition to the limitation of the validated tools used to evaluate preoperative anxiety in children, mainly limited to the Yale Preoperative Anxiety Scale, which covers precisely these age groups.

Children undergoing emergency surgery; who received pre-anesthetic medications before or during music listening; and/or those with hearing or cognitive problems reported by the parents/guardians of the child were excluded. It is noteworthy that all participating children were hospitalized on the same day of surgery and subject to general anesthesia.

For sample size calculation, given the scarcity of published data regarding the variables of interest, it was used the Central Limit Theorem, which ensures that the distribution of sample means derived from samples larger or equal to 30 approximates the normal distribution for any population^9^.

Data collection was performed in the waiting room of the Pediatric Surgical Unit and began by gaining the consent of the parents/guardians through signing the Informed Consent Form, and by a drawing by pre-school children, in the space dedicated to the Free Informed Assent Form (IAF); and, in the case of school-age children, by signing the IAF. It is noteworthy that throughout data collection, parents/guardians were present in the room. 

The participating children were randomly allocated by means of a list of random numbers generated by computer, into two groups: experimental (EG) and control (CG), with allocation rate of 1:1. After randomization, the clinical and demographic data such as age, gender, type of surgery, previous surgical procedures and degree of relatedness of the accompanying persons in each group were registered.

Regarding the participants allocated in the CG, the research team measured and recorded the physiological and behavioral variables of anxiety (basal time) and then waited for 15 minutes - a period in which children were not subject to any intervention by the research team, and they were observed only in relation to the conventional care of the Pediatric Surgical Unit. After the 15 minutes interval, the physiological and behavioral variables of anxiety were measured and recorded again (post-intervention period).

The conventional care of the Pediatric Surgical Unit consisted in providing some toys and a TV, as well as the presence of the family and other children who were also awaiting surgery. All children participating in this study, both in the CG and EG were exposed to conventional care and these distracting factors were not controlled in this study, except in the EG, in which the participating children were also exposed to music listening for a specific period of 15 minutes.

As for the children allocated in the EG, the research team measured and recorded the physiological and behavioral indicators of anxiety (basal time). Subsequently, the research team provided four pre-selected songs for participants to listen using a *MP3 Player* device for 15 minutes, at which time they received no clinical intervention and were subjected to music listening, the independent variable of this research. After the 15-minute period, the research team measured and recorded the physiological indicators and behavioral manifestations of anxiety again (post-intervention period).

The songs that comprised the repertoire were instrumental, non-lyrical, with 60-80 beats per minute, with sound level of 60 dB and low tones, performed using stringed instruments and with minimal percussion, in accordance with the recommendations of the *Joanna Briggs* Institute^10^. There were two Brazilian children's songs - *O cravo brigou com a rosa* and *A canoa virou*, tracks of the album called *Cantigas de ninar*, interpreted by Alexandre Guerra and Michel Freidenson - and two American folk songs, *Over the Rainbow*, track of the album *Bebé: Nanas y canciones infantiles para la relajación del bebé*, and *Amazing Grace*, track of the album *Taught me love* of *Trevor Johan Binkle*, performed in the child's order of preference and provided in a *Sony NWZ-B172F* MP3 player with disposable *Bright 0025* in-ear headphones or *Acorde SH-S1 multimedia MP3 headphones*, used mainly for children with small outer ear. After each use, the headphones were cleaned with 70% alcohol, according to standard precautions in audiology^11^.

The choice of the 15 minutes duration period for the music listening was based on the average duration indicated by studies carried out in adult populations, varying from 15 to 30 minutes^10^ or from 20 to 30 minutes^12^, since there was no strong evidence indicating the minimum time needed.

Anxiety, the dependent variable of this study, was assessed using indicators of physiological and behavioral variables. The indicators of physiological variables were heart rate (HR), respiratory rate (RR), blood pressure (BP) and oxygen saturation (SpO2). BP and HR were measured using the automatic *Omron HEM-710INT* blood pressure monitor and the *Omron H003DS* small adult blood pressure cuff; and, in the cases of children with arm circumference higher than 23 cm, it was used the *Omron HEM-CR24* cuff. A pediatric fingertip oximeter, *PM100D New Tech*, was used to measure the SpO_2_. The RR, in turn, was measured by observing the number of breaths/min.

In order to assess the behavioral variables of anxiety, it was used the modified Yale Preoperative Anxiety Scale (m-YPAS), an instrument validated and translated in Brazil, which has been widely used in international^13^ ^-^ ^14^ and national^(2, 15)^ studies to measure the anxiety levels in children, especially in the immediate pre-anesthetic period and in the anesthetic induction moment. The m-YPAS consists of an observational scale composed of twenty-two categories divided into five areas: activities, apparent awakening state, vocalization, emotional expression and interaction with family^2^ ^,^ ^15^.

All domains have four categories with the following scores: category 1 (0.25); category 2 (0.50), category 3 (0.75) and category 4 (1.00). The only exception is the vocalization domain, which presents six categories with scores distributed as follows: category 1 (0.17); category 2 (0.33); category 3 (0.50); category 4 (0.67); category 5 (0.83) and category 6 (1.00). For the final score, the score of the category that best describes the observed behavior is assigned to each domain, added up and the result is multiplied by 20. The minimum score is 23.4 and the maximum is 100 points, where the scores in the range of 23.4 to 30 points do not indicate anxiety, and scores higher than 30 points indicate state of anxiety^2^.

As for the blinding, children were masked in relation to the musical intervention by adapting the strategy used in an Australian study^16^, in which the participants in the control group also listened to music, but only after the completion of data collection. As in that study, children were not told which group they belonged, but they knew they would listen to music. Although the research team was not masked and knew who belonged to the CG and EG, the children did not know how distinguish because all listened to music. In the case of CG, the researcher provided MP3 Players for children to listen to the music at the time that the measurements had been completed, that is, after the two measurements of physiological and behavioral variables of anxiety.

The research team was composed of the lead researcher and two undergraduate Nursing students. The students received prior training for the application and completion of the m-YPAS and the proper handling of the equipment used for measuring the physiological variables, but no interobserver reliability analysis was carried out during training and data collection.

In addition to the participants, the statistical staff was also masked, since before the data be available, the groups CG and EG were coded as G1 and G2 to prevent the statisticians from distinguishing the group receiving the intervention. Thus, although the research team was not blinded, yet this study is characterized as double-blind, as the participants and statistical staff were blinded.

The collected data were coded and double entered into *Excel* spreadsheets, *Microsoft Office 2010*, and exported to *The SAS System Software*, version 9.0, for the analyzes. In addition to the descriptive analysis (mean, standard deviation and frequency), for the inferential analysis, it was applied the Chi-Square test of Independence, Mann-Whitney test and Analysis of Variance (ANOVA) with repeated measurements followed by Tukey test for multiple comparisons. The level of significance utilized in all tests was 5%.

## Results

**Figure 1:**
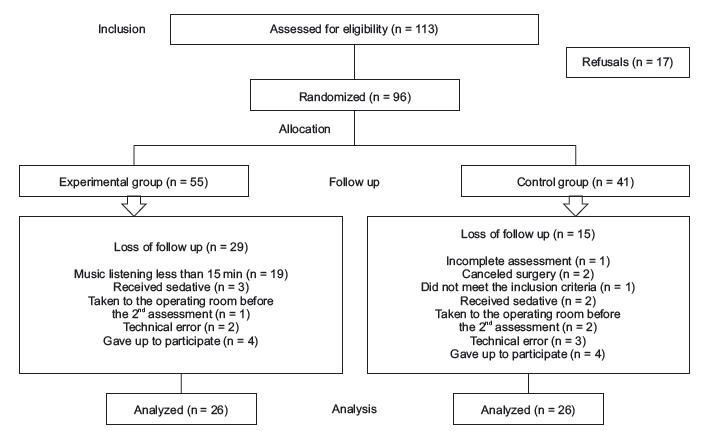
Research flowchart. Brasilia, DF, Brazil, 2015

Of the 113 participants assessed for eligibility, 17 refused to participate and 96 were randomly allocated to the EG or the CG, and only 52 children were included in the final sample, as detailed in Figure 1.

Regarding the clinical and demographic characteristics of participants, there was no statistically significant difference between the groups (Table 1).

**Table 1:**
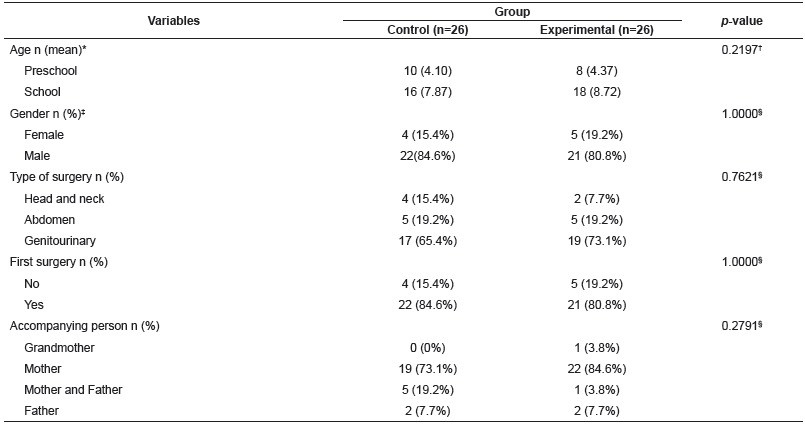
Clinical and demographic data of participants. Brasilia, DF, Brazil, 2015

* n (mean), absolute number and sample mean; ^†^ *p*-value, significance level of the Mann-Whitney test; *‡* n (%), absolute number and percentage of the sample; ^§^ *p*-value, significance level of the Chi-Square test of Independence

Regarding the classification of surgical procedures, it is highlighted the genitourinary surgical procedures which include postectomy, orchidopexy, inguinal hernia repair, hypospadias repair or hydrocele repair and complete penile plastic surgery. The surgeries in the abdominal region consisted of herniorraphy/hernioplasty and videolaparoscopy. The head and neck surgeries, in turn, comprised amygdalectomy, adenoidectomy, incision and drainage of the lingual and sublingual abscess, excision and suturing of cervical lymphangioma, exeresis of thyroglossal cyst and branchial cyst.

**Figure 2:**
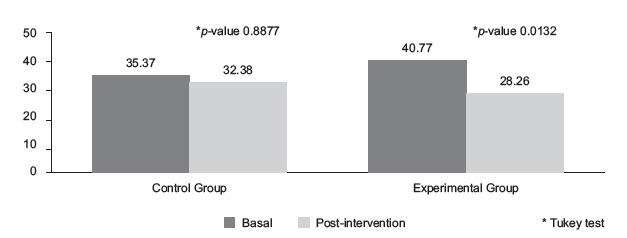
Mean of the modified Yale Preoperative Anxiety Scale scores, according to

Regarding the mean of the m-YPAS scores, there was a statistically significant effect in the interaction between group and time (*p* = 0.0441), analyzed by ANOVA. Based on the Tukey multiple comparison test, it was noted that within the EG, the mean of the m-YPAS scores was statistically different in the two times, unlike in the CG which showed a *p*-value = 0.8877 (Figure 2).

As regards the domains of the m-YPAS, there was a statistically significant effect of the interaction between group and time in relation to four domains, with the exception of the domain of interaction with relatives. Based on the Tukey multiple comparison test, it was found that such statistical significance was related only to the EG (Table 2).

**Table 2:**
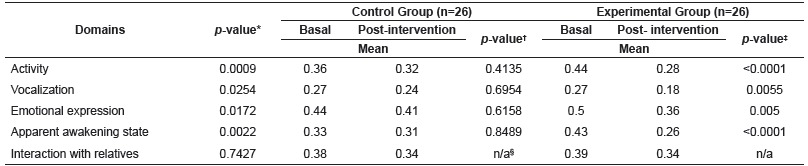
Mean of the scores of the modified Yale Preoperative Anxiety Scale domains, by group for each assessment time. Brasilia, DF, Brazil, 2015

* *p*-value, ANOVA with repeated measurements over time; ^†^ *p*-value, Tukey multiple comparisons for the CG; ^‡^ *p*-value, Tukey multiple comparisons for the EG; ^§^ n/a, not applicable to Tukey test, since ANOVA *p*-value was not significant (0.7427)

In the analysis of physiological variables, the pre-school and school ages were considered separately, since each age group has a different average for the physiological variables. It was found, by analysis of variance (ANOVA), that there was a statistically significant difference of triple interaction among the variables age group, group and time on the heart and respiratory rates (Table 3).

**Table 3:**
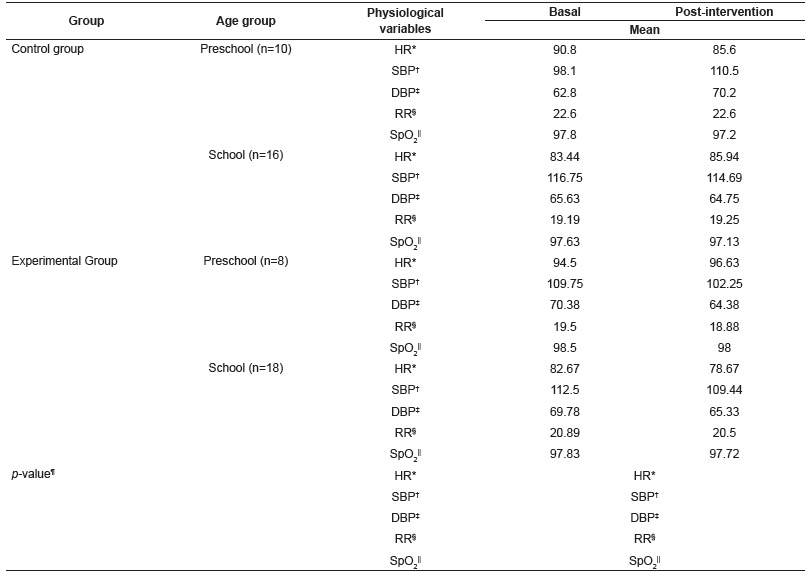
Mean of the physiological variables by group, age and time. Brasilia, DF, Brazil, 2015

* HR, heart rate; ^†^ SBP, systolic blood pressure; ^‡^ DBP, diastolic blood pressure; ^§^ RR, respiratory rate; ^||^SpO_2_, oxygen saturation; ^¶^ *p*-value, ANOVA with repeated measurements over time

In view of the above, the Tukey multiple comparisons test was carried out and it identified that in the EG, school age participants had lower average heart rate in the post-intervention time compared to pre-school age participants (*p* = 0.0101). In relation to the respiratory rate, by Tukey test, it was found that in the CG, preschool children had higher average respiratory rate than schoolchildren in both times, basal and post-intervention (*p* = 0.0312 and *p* = 0.0344). It was also detected, by Tukey test, intergroup differences, considering that pre-school children from the EG had lower average respiratory rate in the post-intervention time compared to pre-school children of the CG (*p* = 0.0453).

## Discussion

In this study there was a predominance of males and genitourinary surgeries, which is justified in that they comprise 60% of the pediatric surgeries demand^17^, followed by phimosis, hypospadias, hydrocele and cryptorchism because they are surgical conditions performed only in male. The mother was the child's main accompanying person in both groups, fact widely described in the pediatric literature^2^.

A sample loss rate was 54%, which contrasts with the average dropout rate in studies related to musical intervention in adults^12^, comprising 0-13% of the total sample. One of the main causes of sample losses, particularly in the EG, was the interruption of the music listening due to the musical style and duration of the intervention.

Refusals to participate because of the musical style were also found in a study using classical music to children in the postoperative period^7^. Some studies point out that the musical preferences of participants must be met, considering that there is a greater impact and a higher correlation with the degree of relaxation^12^ ^,^ ^18^, and songs considered stimulating may also be used^18^. In this way, these studies question the music considered as sedating and attribute the effects of music not only to the structural characteristics of the musical work, but also to the extramusical aspects such as preference, culture and emotions of the listener. The time set at 15 minutes for the intervention, even being smaller in comparasion with other studies in a systematic review^6^ that reported a duration of 30 and 45 minutes, was not supported by all, considering that the average time tolerated by the dropouts was 8 minutes. Such studies, however, were conducted in the immediate postoperative period, which may have favored acceptance of the intervention, the only distraction factor at that time, considering that children had restricted mobility and were under anesthesia, unlike children in this study who had several options like toys and the presence of family members and other children in the waiting room.

The other losses were related to the service routine, as routing to the surgical center before completing the intervention and administration of sedatives, reasons that are similar to those described in Cochrane's systematic review^12^. This indicates the need to develop more clinical trials with hybrid research designs, which simultaneously assess the effect of the intervention under study and the implementation of this strategy in clinical practice^19^, since the healthcare services are dynamic and "difficult-to-monitor" scenarios.

On analyzing the mean of the m-YPAS scores, it was noted that it was lower in the EG in the two times (*p* = 0.0132), which did not occur in the CG. The reduction of almost 31% in the m-YPAS scores, from 40.77 at the basal time to 28.26 in the post-intervention time, is related to the music listening, since this was the only intervention performed in the EG during this time interval, thus corroborating the studies that also found statistical significance for the group-time interaction^13^ ^-^ ^14^. Despite the statistical significance, this result should be interpreted with caution, since the loss of follow-up in this group was high, taking into account that 34.5% of participants did not tolerate the duration set for the intervention.

It is also highlighted the domains of Activity, Vocalization, Emotional expression and Apparent awakening state, which showed a statistically significant reduction in the mean scores of the EG in the two time points (basal and post-intervention). The only domain that did not reach statistical significance was the domain of Interaction with relatives, in which the scores of both groups were between 0.39 and 0.34 points, indicating less behavioral change. This result can be explained by the fact that children had the presence of the accompanying person while they were in the waiting room.

As for the physiological variables, statistically significant intragroup differences indicate, at first, only physiological evidences, since it is expected that preschool children present higher HR and RR than schoolchildren. However, in the intergroup analysis, the significant decrease in the RR between preschool children of the EG and preschool children of the CG in the post-intervention time (*p* = 0.0453) points out the musical intervention as a differentiating factor between the two groups, corroborating other studies^7^ ^,^ ^13^. It is worth mentioning that this research is one of the few that considered the specificity of age range in the analysis of physiological variables, as in most studies, the analysis included participants of different ages, without considering physiological values, according to pediatric age groups^7^ ^,^ ^13^.

Among the limitations of this research, it is mentioned the reduced size of the sample and the absence of an inter-observer reliability analysis in the application of the m-YPAS, aspects that may cause selection bias and external validity bias. In addition, the selected musical genre, combined with the expanded age range of participants, which ranged from 3 to 12 years, and the duration of the intervention contributed to the high dropout rate among participants allocated in the EG. It is suggested that future studies include narrower age groups, considering that the musical preferences may change with age, compare the selection of sedative songs with the musical preferences of children, and investigate the effects of music listening with a duration of less than 15 minutes.

Furthermore, for being a research that uses music, it is important that the children's hearing conditions are assessed more accurately, based not only on the reports of the child's parents/guardians, but also in simple tests used in nursing to assess hearing acuity, such as the whisper test, which would require more time for data collection.

It is worth mentioning that this pilot study was conducted with high methodological rigour, based on the CONSORT guidelines, which enables the replication of this study and the use of its findings in future systematic reviews. In addition, it subsidizes new studies that allow the incorporation of music listening as an evidence-based nursing intervention in the care of children undergoing surgical procedures.

## Conclusion

There was a statistically significant difference between the groups regarding preoperative anxiety only in the physiological variable because the respiratory rate of preschool children in the experimental group reduced in the second measurement compared to the control group. Regarding the behavioral variable of anxiety, the experimental group showed a statistically significant reduction in the anxiety levels after 15 minutes of music listening, specifically regarding the behavioral domains of activity, vocalization, emotional expression and apparent awakening state. The music listening, therefore, emerges as a potential resource in nursing care to assist in the relief of preoperative anxiety in children undergoing surgical procedures.
